# Use of PtC Nanotips for Low-Voltage Quantum Tunneling Applications

**DOI:** 10.3390/mi13071019

**Published:** 2022-06-28

**Authors:** Michael Haub, Thomas Guenther, Martin Bogner, André Zimmermann

**Affiliations:** 1 Institute for Micro Integration (IFM), University of Stuttgart, Allmandring 9b, 70569 Stuttgart, Germany; thomas.guenther@ifm.uni-stuttgart.de (T.G.); martin.bogner@gmx.de (M.B.); andre.zimmermann@ifm.uni-stuttgart.de (A.Z.); 2 Hahn-Schickard, Allmandring 9b, 70569 Stuttgart, Germany

**Keywords:** quantum tunneling, focused ion beam, FIB, focused electron beam, FEB, EDX, scanning tunneling microscopy, platinum carbon, DLC

## Abstract

The use of focused ion and focused electron beam (FIB/FEB) technology permits the fabrication of micro- and nanometer scale geometries. Therefore, FIB/FEB technology is a favorable technique for preparing TEM lamellae, nanocontacts, or nanowires and repairing electronic circuits. This work investigates FIB/FEB technology as a tool for nanotip fabrication and quantum mechanical tunneling applications at a low tunneling voltage. Using a gas injection system (GIS), the Ga-FIB and FEB technology allows both additive and subtractive fabrication of arbitrary structures. Using energy dispersive X-ray spectroscopy (EDX), resistance measurement (RM), and scanning tunneling microscope (STM)/spectroscopy (STS) methods, the tunneling suitability of the utilized metal–organic material–platinum carbon (PtC) is investigated. Thus, to create electrode tips with radii down to 15 nm, a stable and reproducible process has to be developed. The metal–organic microstructure analysis shows suitable FIB parameters for the tunneling effect at high aperture currents (260 pA, 30 kV). These are required to ensure the suitability of the electrodes for the tunneling effect by an increased platinum content (EDX), a low resistivity (RM), and a small band gap (STM). The STM application allows the imaging of highly oriented pyrolytic graphite (HOPG) layers and demonstrates the tunneling suitability of PtC electrodes based on high FIB aperture currents and a low tunneling voltage.

## 1. Introduction

The use of the quantum mechanical tunneling effect as a transducer principle for sensor applications enables sensor signals of very high resolution [1,2,3,4,5,6,7,8,9,10,11]. Furthermore, a significant miniaturization of the sensor core area [12,13] is possible. This is due to the high sensitivity of the tunneling effect, which is a result of its exponential dependence on the tunneling current and electrode spacing. Theoretically, the tunneling current changes by an order of magnitude for a change in distance of a few Å. This allows accelerometers to measure the smallest accelerations or extremely reduce the seismic mass to detect the smallest deflections. For the latter, the acceleration equivalent noise is the limit for miniaturization. Therefore, to implement the required tunneling electrodes, the fabrication of very fine electrode tips is necessary to keep attractive forces low at tunneling spacings of a few nm [13]. If the radii of the tips are too large, there is a risk of a snap-in effect when they approach each other. Additionally, the common process of etching a tungsten tip regarding the STM observation, the method on hand allows for the integration into sensors with movable parts. Thus, using a dual beam consisting of Ga-FIB and FEB in combination with a GIS is suitable for tip production. In addition to the tip radii, the electrical potential between the tunneling electrodes also significantly influences the magnitude of attractive forces. Therefore, tunneling applications with freely movable electrodes aim to achieve the lowest possible tunneling voltages [13]. Through the precursor used in this work (MeCpPtMe_3_ = trimethyl(methylcyclopentadienyl)platinum(IV)), a metal–organic material is formed after deposition, consisting of platinum (Pt) particles embedded in a diamond-like carbon (DLC) matrix. Moreover, it is known that the conduction mechanism of metal–organic materials is based on intrinsic tunneling between the metallic grains [14,15,16,17,18,19,20]. Thus, depending on the microstructure, changes in the resistance of a nanowire can be caused and measured using small strains, as demonstrated by Schwalb et al. [21]. If tunneling is to be accomplished using an additional vacuum gap, it must be ensured that the platinum particles are located in the edge region of the electrode tips. Consequently, if the distance between the platinum particles is too large, the tunneling effect comes to a standstill. Therefore, the present study aims at investigating whether the FIB/FEB parameters influence the properties of the resulting material and whether the material is suitable for electron tunneling at small tunneling voltages regarding the material proportions, the material resistivity, and the band gap. Thus, the methods of EDX and RM are used. In addition, the PtC tips are applied to a HOPG sample in an STM. An HRTEM (high-resolution transmission electron microscopy) analysis about PtC nanotips is extensively discussed elsewhere by Haub et al. [22]. The study comparing several PtC tips by FIBID and FEBID using HRTEM analysis by the authors shows significant differences in the nanostructure regarding the location, size, and distribution of the Pt particles.

## 2. Materials and Methods

**Figure 1:**
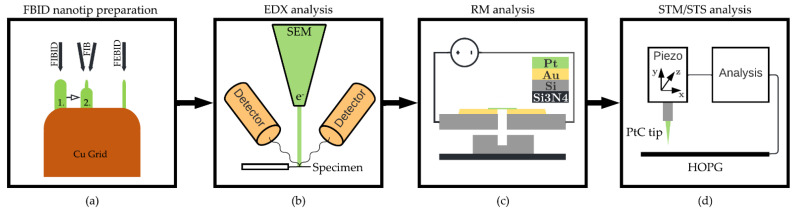
Overview of the applied methods. (**a**) Principle of one-step FEBID and two-step FIBID nanotip preparation. (**b**) Principle of SEM-based EDX analysis. (**c**) Principle of analyzing PtC resistivity by deposition and patterning of nanowires to a PolySi beam on a MEMS chip. (**d**) Principle of STM/STS analysis by deposition of PtC nanotips to a PtIr wire.

Before investigating PtC tips, a suitable process must be developed to generate tips with radii of a few nm. This is based on a multistep process involving focused electron beam deposition (FEBID) and focused ion beam deposition (FIBID), as well as subsequent patterning by the ion beam (Figure 1a). For EDX analysis (Figure 1b), the PtC nanotips must be deposited on an Omniprobe lift-out grid to ensure compatibility with the Thermo Fisher Osiris ChemiStem (Waltham, MA, United States, SEM-based EDX). In addition, the resistivity of free-standing PtC nanowires fabricated with FEBID/FIBID is measured (Figure 1c) using a Tektronix Keithley 2614B (Beaverton, OR, United States) SMU (source measuring unit). For tunneling spectroscopy measurement with a Nanosurf NaioSTM, the PtC tips are deposited on a platinum iridium (PtIr) wire (Figure 1d). The STM is also used to apply tunneling electrodes and prove tunneling suitability by imaging a graphene layer. Meanwhile, the polysilicon (PolySi) structures that serve as the basis for the resistivity measurement are fabricated using the PolyMUMPs process from MEMSCAP Inc. Thus, before describing the methods in detail, a brief overview of previously published results on PtC is provided.

### 2.1. Platinum Carbon Composite

This investigation is based on the use of a metal–organic precursor material, MeCpPtMe_3_. In the deposited metal–organic composite by FEBID/FIBID, the metallic components are present in a diamond-like amorphous carbon (DLC) matrix, consisting of an sp^2^ (graphite) and an sp^3^ (diamond) bonding structure. This is shown by Matsui et al. [23], Kanda et al. [24], and Stanishevsky et al. [25] by Raman spectroscopy; by Fernández-Pacheco et al. [16] by X-ray photoelectron spectroscopy (XPS); and by Frabboni et al. [26] by electron energy loss spectroscopy (EELS). The comparison of the resistance of bulk Pt and PtC has also been widely investigated and can be confirmed by previous studies [17,27,28,29,30,31,32,33,34]. PtC has a high material resistance because of the contamination by the high ohmic DLC. The dependencies regarding deposition parameters [17,34,35,36,37,38], temperature [29,34,39], technology (FIB/FEB) [34,37], percolation of gallium [40], or material thickness [16,29] are deeply investigated. In particular, Tao et al. [38] presented the lowest resistivity of the deposited nanowires at the highest ion current of 222 pA. The resistivity of FIBID and FEBID Pt is about one to two orders of magnitude higher for FIBID Pt and about four to five orders for FEBID Pt compared with the resistivity of bulk Pt [16,29,34,38]. Moreover Utke et al. [41] gave a comprehensive review about mechanical properties of three-dimensional nanostructures obtained by FEBID/FIBID. Depending on the used technology (FEBID: approx. 10–100 GPa, and FIBID: approx. 100–150 GPa), there is a wide range of measured Young’s modulus based on pillar bending, pillar compression, and nanoindentation for the MeCpPtMe_3_ precursor. Furthermore, the hardness investigated by Reiser et al. [42] through nanoindentation (material resistance to localized plastic deformation) is dominated by the carbonaceous material with approximately 6 GPa for FEBID PtC and 9 GPa for FIBID PtC and higher than for bulk Pt.

Regarding the tunneling effect, the nanostructure of the PtC composite aggravates electron tunneling because of the high ohmic parts of DLC. The tunneling effect is primarily used in a scanning tunneling microscope (STM) for commercial purposes with pure metal tips with low resistance. This enables tunneling currents of up to 100 nA. However, to determine the suitability of the tunneling mechanism with PtC tips, the nanostructure has to be investigated. In order to use PtC tips for tunneling through a vacuum gap, a sufficient amount of Pt in the nanostructure is necessary to achieve a high probability of Pt particles located at the edge of the tips. Previous publications have described the conduction mechanism of metal–organic or granular materials as intergrain tunneling between the metallic components [14,15,16,17,18,19]. For example, Samanta et al. [19] demonstrated intergrain quantum tunneling in a FIBID PtC nanowire and showed the dependence of the electrical transport properties and a pressure-induced process. Moreover Kolb et al. [20] showed the variability of the tunneling properties in FEBID-based PtC wires depending on the physisorption of polar gas molecules. In the present work, a vacuum gap is added as an additional tunneling component to show the suitability of PtC nanotips for vacuum tunneling applications at low tunneling bias.

### 2.2. Energy Dispersive X-ray Spectroscopy

**Figure 2:**
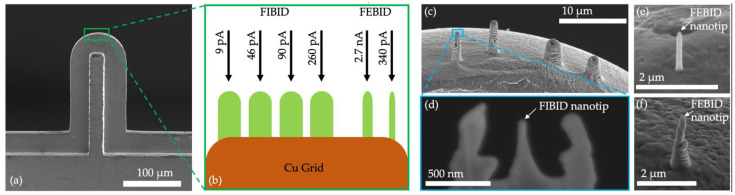
Process of preparing PtC nanotips for EDX analysis with (**a**) a Omniprobe lift-out Cu grid and (**b**) the principle of depositing PtC bumps/tips by FIBID/FEBID, (**c**) the SEM image of the deposited PtC bumps by FIBID, (**d**) the SEM detail image of a final FIBID nanotip, (**e**) the 340 pA and (**f**) the 2.7 nA FEBID nanotip deposited without additional patterning.

This study elaborates on the differences between the tips when ionic and electron currents are utilized. As shown in Figure 2a, an Omniprobe lift-out Cu (copper) grid is used to deposit several tips based on FIBID and FEBID. Unlike FEBID or helium-FIBID [43,44,45,46,47], Ga-FIB deposition cannot directly fabricate sharp tips with a thickness of a few tens of nanometers. For the EDX analysis, this is basically not of importance; however, for the later investigation by means of STM analysis, comparable geometries should be produced in order to compare the results. Thus, the preparation of the FIBID tips is based on a multistep process: First, PtC bumps are deposited with ion beam currents of 9, 46, 90, and 260 pA (Figure 2b,c). Second, the bumps are sharpened by the FIB. Third, the PtC geometries are formed into a nanotip with the smallest possible radius by a minimum ion current of 1.5 pA (Figure 2d). The accelerating voltage of the FIB is set to 30 kV. As shown in Figure 2e,f, the FEBID tips are directly deposited without further processing. The accelerating voltage of the FEB is set to 5 kV, and the selected electron beam currents are 340 pA and 2.7 nA.

For the application of the tunneling effect, metallic tips are usually used due to their suitable densities of states of the free electrons. However, the number of free electrons or unoccupied states is significantly lower in the case of a semiconductor or insulator. As a result, higher tunneling voltages must be applied to generate a sufficient tunneling current. The elements in the material are determined by the EDX analysis in order to perform an estimation of the tunneling capability of the material. Since a metal–organic material composite is deposited, the analysis of the respective atomic fractions is worthwhile in the case of this study. Previous publications show considerable deviations with respect to the material fractions of PtC structures due to the use of different equipment and deposition conditions as well as parameters of the EDX analysis (see Section 2.1). Furthermore, it is of particular interest to determine whether there is a difference in the material fractions depending on the technology used (FEB/FIB) and deposition parameters (aperture currents). Here, the probability of platinum particles’ occurrence in the edge region of the tunneling electrodes is significant since these particles contribute significantly to electron tunneling. Thus, the higher the atomic fraction, the higher the tunneling capability of the electrode tips.

**Figure 3:**
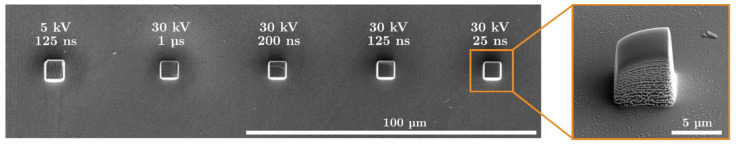
Illustration of five FIBID pads, each with a dimension of about 5 × 5 × 5 µm^3^. On the one hand, the dwell time is varied between 25 ns and 1 µs, and on the other hand, the acceleration voltage is set to 5 or 30 kV.

In addition, the FEB/FIB allows for the modification of the acceleration voltage parameters and the dwell time per grid point. Therefore, for further investigation of the material, several PtC pads are deposited based on different acceleration voltages and dwell times using the ion beam. The pads are deposited on a glass substrate sputtered with gold. The lateral extent of the pads is 5 × 5 µm^2^, with a thickness of about 5 µm. Therefore, especially for the thickness of the samples, the information depth of the EDX analysis has to be considered. This varies depending on the material and is in the order of a few µm. Figure 3 shows the pads deposited in each case and, as an example, a detailed image in the side view on the left. An ion current of 260 pA is maintained at an accelerating voltage of 30 kV, and the dwell time is varied between 25 ns and 1 µs. In addition, a PtC pad is deposited with an accelerating voltage of 5 kV.

The analysis of the atomic fractions in the PtC structure is of great importance for the tunneling suitability and fabrication of the electrode tip. If, for example, an increasing aperture current of the electron or ion beam shows an increasing platinum content in the PtC structure, a higher probability for the occurrence of platinum particles in the edge region of the tip can be derived from this. This has a direct influence on the tunneling effect. According to the literature (see Section 2.1), a significant difference in the elemental compositions of the PtC structures is to be expected due to the electron and ion beam. However, since the proportions also vary between the devices used, an investigation specifically into the dual beam used is necessary. Therefore, the EDX analysis should confirm the literature values, on the one hand, and provide discrete values for the deposited PtC structures, on the other.

### 2.3. Resistivity Measurement

This investigation determines the resistivity of the PtC composite. Moreover, it is already known from the literature that, depending on the beam used (FEB or FIB), the deposited material has different resistivity values. Therefore, since the material resistivity directly influences the tunneling effect and the measurement signal, it has to be investigated to characterize the tunneling current measurement. Ultimately, the resulting values are compared with the literature value of pure platinum with a resistivity ρ_PtC_ of 0.105 Ω·µm [48] at room temperature. Accordingly, the literature indicates that the resistivity of FEBID- and FIBID-Pt is four to five orders of magnitude and one to two orders of magnitude higher, respectively (see Section 2.1). The resistivity measurement should show this deviation due to contamination by carbon. Furthermore, the resistivity value has a decisive influence with regard to the geometry of the electrode tips. When tips with radii of a few nm are produced, the resistance, which is in series with the tunneling resistance, increases significantly. This can lead to a limitation of the measurement signal. For this reason, the PtC structure must be characterized according to its resistivity. Furthermore, this investigation compares the material resistance depending on the technology used (FEB or FIB) and the aperture current to deepen the EDX analysis results.

**Figure 4:**
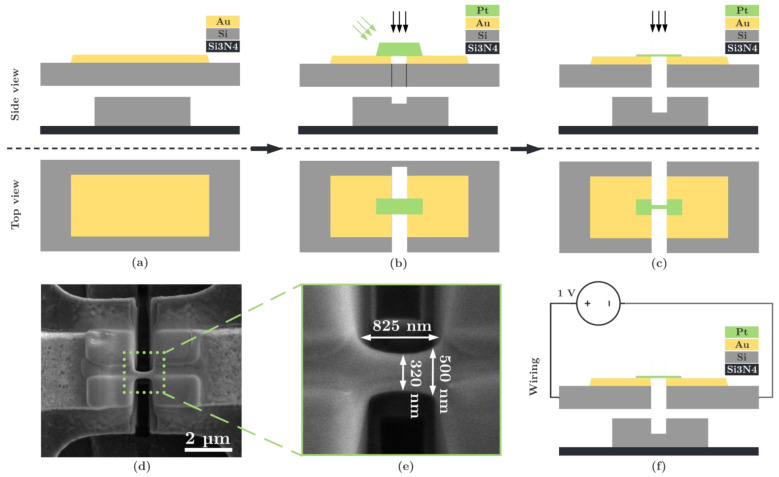
Sample preparation for measuring the material resistance of the PtC nanobeams. Top: principle of fabrication: (**a**) initial state of a free-standing PolySi structure (thickness = 1.5 μm) with a gold pad, (**b**) FIB cut of the PolySi structure and subsequent deposition of a PtC bump, (**c**) patterning of the PtC bump and subsequent final separation of the PolySi structure. The underlying PolySi pad (thickness = 2 μm) serves as protection against unwanted vias to the silicon nitride (Si_3_N_4_) layer and the chip substrate. Bottom: (**d**) SEM image of the final PtC nanowire and (**e**) detailed image with exemplary geometric dimensions. (**f**) Principle of wiring.

To measure the resistance, free-standing PtC nanobeams of similar geometric dimensions are fabricated. The fabrication is performed directly on a MEMS chip on free-standing PolySi structures. The MEMS chip is bonded by a Wire Bonder G5 Single (F&K Delvotec) to a printed circuit board (PCB) to enable the electrical junction. Figure 4 shows the principle and an illustration of a PtC nanobeam. A total of seven PtC nanobeams are fabricated based on FEBID, with aperture currents of 43, 340, and 2.7 nA, and based on FIBID, with aperture currents of 9, 46, 90, and 260 pA.

The initial state of a free-standing PolySi structure (thickness = 1.5 μm) with a gold pad is shown in Figure 4a and fabricated using the PolyMUMPs process from MEMSCAP Inc. Before depositing a PtC bump, the PolySi structure is prepared by a FIB cut to enable two separated beams. A small PolySi bridge is left over to ensure the stability of the beams (Figure 4b). The next step is to pattern the PtC bump and the subsequent final separation of the PolySi structure (Figure 4c). The underlying PolySi pad (thickness = 2 μm) in Figure 4a–c serves as protection against unwanted vias to the silicon nitride (Si_3_N_4_) layer and the chip substrate. Figure 4d shows a SEM image of a final PtC nanowire, and Figure 4e a detailed image with exemplary geometric dimensions. Using a Keithley 2614B SMU, an electrical voltage of 1 V is applied to the PtC beams (Figure 4f), and the resistance is measured on this basis. The resulting resistance is subtracted from the resistance of the leads, which is measured prior to the fabrication of the beams. Based on the resistance measurement, the resistivity can then be calculated via the geometric parameters according to:

(1) $$\rho_{PtC}=\left(R_{total}-R_{W}\right)\;\frac{A_{PtC}}{l_{PtC}}$$

with the resistivity *ρ_PtC_*, the total resistance *R_total_*, the resistance of the wiring *R_w_*, the cross-sectional area *A_PtC_*, and the length *l_PtC_* of the PtC nanowire.

### 2.4. Scanning Tunneling Microscopy

This study aims to investigate the possibility of using PtC tips in quantum tunneling vacuum gap applications at a low voltage range of a few hundred mV. Consequently, the EDX and RM analysis results will be validated on an STM using a HOPG probe. After deposition and patterning of the nanotip, the tunneling effect can be proven by measuring the tunneling current between a PtC electrode and a HOPG probe at distances of some nm.

The atomic structures of a surface can be imaged with an STM [49]. Here, both tunneling electrodes are fixed. That is, one of the electrodes is moved via piezo actuators in the x, y, and z directions to control the sample’s distance and scan it. In this way, a line-by-line image of the sample is generated. In this investigation, the NaioSTM tunneling microscope from the Nanosurf company [50] was used. Besides, the measurement or imaging of a HOPG sample allows reproducible reference measurements due to the favorable order of the atoms and represents a useful procedure for testing the tunneling suitability of electrode tips.

The STM allows the PtC electrodes to be specifically tested for their tunneling suitability. The PtC electrodes are fixed so that the risk of snap-in effects can be excluded [13]. As a result, there is a large degree of independence between the selected tunneling voltage and the active area of the electrode tip with regard to attractive forces. Consequently, independent of the constraints imposed by the geometry, it is possible to obtain discrete conclusions about the electronic character in terms of tunneling suitability. The goal of the analysis is, first, to image the HOPG sample and, second, to measure the band gap. However, imaging of the sample can only be realized when the band gap or the DLC edge layer is overcome. Therefore, a minimum voltage must be selected at the tunneling electrodes. The band gap can be measured by tunneling spectroscopy. In this case, the electrode is moved close to the HOPG sample until the tunneling effect occurs. The tunneling voltage is then varied over a defined range. The resulting I/U curve shows the voltage range in which the tunneling current comes to a standstill, marking the band gap of the PtC compound at the edge of the electrode tip. Thus, the HOPG sample can then be imaged by selecting a sufficient tunneling voltage. This serves as a particularly meaningful reference for the tunneling capability of the PtC electrode tip at low tunneling voltages since the imaging of the atomic structure is only possible via the occurring tunneling effect. The analysis aims to compare the measured values based on electrode tips fabricated by choosing different parameters of an electron and an ion beam.

**Figure 5:**
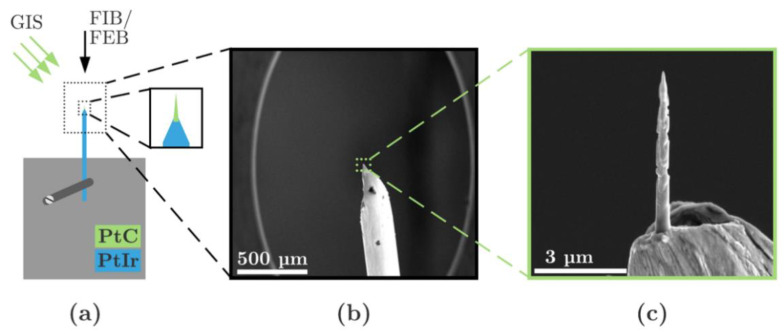
Sample preparation for measurement of PtC electrodes by scanning tunneling microscopy. (**a**) Principle of sample preparation with electron and ion beam as well as GIS. The PtIr wire is clamped to a sample holder. (**b**) SEM image of the PtIr wire. (**c**) A detailed image of the PtC tip on the torn PtIr wire end.

Additionally, in NaioSTM, PtIr electrodes are preferably used because they are not subject to significant oxidation. However, due to the brittleness of the PtIr material, the electrodes can be fabricated by a wire-tearing process. Subsequently, the PtC electrodes are deposited on the PtIr wire tip using the FIB/FEB and GIS. Figure 5 shows the principle of sample preparation and an example of a PtC electrode (diameter at the anchor point ≈ 500 nm) on a PtIr wire tip (diameter ≈ 250 µm) in detail. According to the EDX analysis findings, three PtC tips were prepared using FEB (340 pA) and FIB (9 and 260 pA).

In the STM, the HOPG sample is positioned and the PtIr wire is clamped. After rough approaching, the electrode is automatically approached to the sample using a stick-slip and a piezo actuator until the tunneling effect occurs. Tunneling spectroscopy is now performed to measure the band gap or a suitable tunneling voltage. The imaging of the sample by a scanning process is then used for further proof of the tunneling effect that occurs. Since sample cleanliness is important in STM imaging, samples can be cleaned via annealing, resistive heating, deionized water, and vacuum treatments of the tip, such as electron or ion bombardment, high-field treatment by raising the tunneling voltage for a short time, or tip indentation into a soft metal [51]. In this work, the original state of the PtC nanotips for tunneling will be investigated. In order to reduce a change of the PtC nanotips to the minimum, the wire tip configuration is mounted into the STM immediately after fabrication, and further treatments of the tips are avoided.

## 3. Results

### 3.1. Energy Dispersive X-ray Spectroscopy

**Figure 6:**
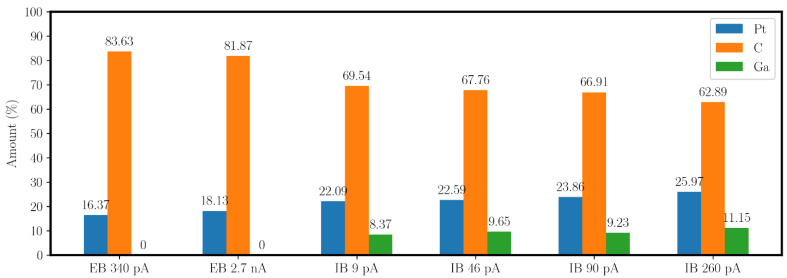
The EDX analysis yields two FEBID tips with 340 pA and 2.7 nA and four FIBID tips with 9, 46, 90, and 260 pA. The FIBID tips each contain a Ga amount, which is not present in the FEBID tips due to the technology.

The results from the EDX measurement of the fabricated PtC tips are shown in Figure 6. In comparison, there are two FEBID tips with 340 pA and 2.7 nA and four FIBID tips with 9, 46, 90, and 260 pA. The FIBID tips each receive a gallium amount of about 10%, which is not present in the FEBID tips due to the technology. In addition, oxygen is detected in all samples, but due to its small amount, it can be neglected in the following presentation and discussion.

Figure 6 demonstrates an increase in platinum content with an increasing aperture current. The FEBID tips show a slightly higher platinum amount of about 2% when the electron current is increased from 340 pA to 2.7 nA. However, currents of 2.7 nA and higher are not practical for the fabrication of PtC nanotips, as this is accompanied by a significant decrease in resolution during deposition. Compared with the FIBID tips, a maximum difference in platinum content of about 10% can be observed for the tips investigated. Due to the additional amount of implanted gallium, the metallic content is about 30–37%, which is 14–20% higher than in the PtC by FEBID. The higher platinum content confirms the assumption made through the TEM analysis that there is a reduced amount of DLC when using the FIB. The platinum content does not change significantly after further EDX analysis to investigate the dependence on dwell time and selected accelerating voltage. However, the proportion of gallium in the microstructure increases overall by about 2.5% when the dwell time is increased from 25 ns to 1 µs since more time is given for the implantation of the Ga+ ions.

For the fabrication of the PtC tunneling electrodes, it can be determined based on this investigation that higher currents lead to an increased platinum content and a lower carbon content. Furthermore, the investigation shows the independence of the accelerating voltage and the dwell time. Moreover, compared with the electron beam, the metallic content increases significantly due to the implantation of Ga+ ions. In summary, the EDX analysis leads to the result that the use of a high ion current is advisable for the highest possible tunnel suitability.

In addition, a study comparing several PtC tips by FIBID and FEBID using HRTEM analysis by Haub et al. [22] leads to the recommendation to use a high current FIBID process to obtain a high tunneling suitability of PtC nanotips. In particular, the FEBID tips show small Pt grains and large spacing between the particles. For FIBID, by increasing the ion current, smaller and more homogeneously distributed particles in the microstructure increase the probability of their occurrence in the edge region and, consequently, show higher suitability for the tunneling effect when two tips approach each other.

### 3.2. Resistivity Measurement

From the measurement of the resistivity, different values result when the aperture currents and the beam used (FEB or FIB) are varied. Table 1 shows geometrical data and the original resistance according to the leads. The resistivity of the PtC results from the total resistance minus the resistance of the wiring and the geometric data of the nanobeams.

**Figure 7:**
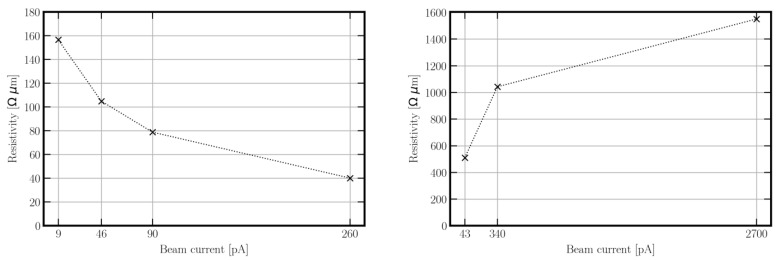
Comparison of resistance measurement of PtC nanowires by FIBID with aperture currents of 9, 46, 90, and 260 pA (**left**) and FEBID with 43, 340, and 2.7 nA (**right**).

**Figure 8:**
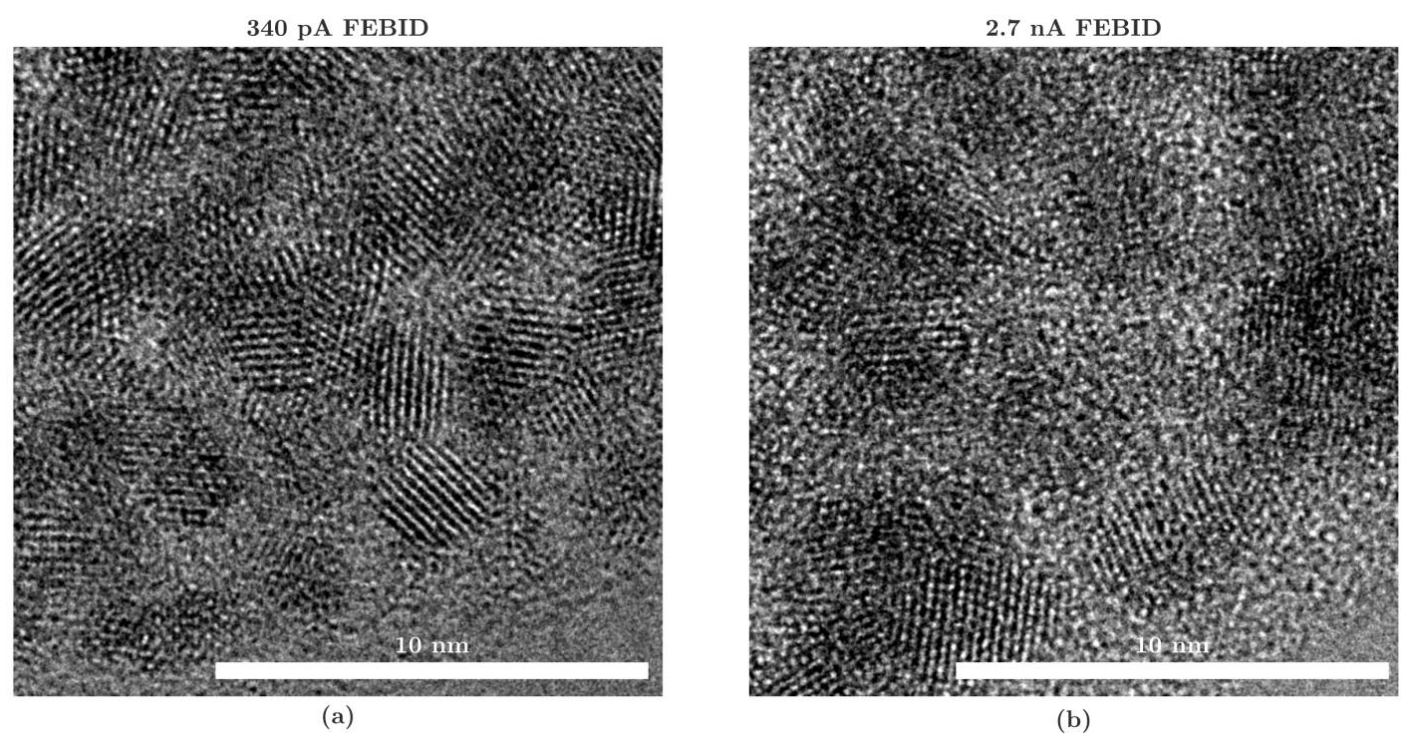
Comparison of the microstructure of (**a**) 340 pA and (**b**) 2.7 nA FEBID PtC. HRTEM images show the edge area of PtC nanotips captured by a Thermo Fisher Titan3. The 340 pA PtC shows smaller grains and higher density of the particles, then the 2.7 nA PtC with larger DLC areas between them.

According to Figure 7, a decrease in resistivity with an increasing aperture current can be seen for the FIBID PtC. Thus, the 9 pA PtC shows a resistivity ρ_PtC_ of 156.61 Ω·µm, that of the 46 pA PtC is 104.93 Ω·µm, that of the 90 pA PtC is 78.86 Ω·µm, and that of the 260 pA PtC is 40.15 Ω·µm. Therefore, in previous investigations related to the present work, the resistivity or conductivity correlated with the proportion, particle size, and distribution of Pt in the PtC structure. Interestingly, the measurements on the FEBID nanobeams show an inverse resistivity behavior. That is, as the aperture current increases, the resistivity of the PtC also increases. Thus, the 43 pA PtC shows a resistivity ρ_PtC_ of 510.69 Ω·µm, that of the 340 pA PtC is 1042.57 Ω·µm, and that of the 2.7 nA PtC is 1551.02 Ω·µm. Additionally, the EDX analysis of the platinum components in the microstructure shows similar values at different electron currents. However, HRTEM analysis (Figure 8) shows an increasing size and lower distribution density of the Pt particles as the aperture current of the FEB increases.

This causes increased resistivity. That is, the size and distribution of Pt particles in the microstructure have a significant effect on the conductivity of the PtC. Thus, with respect to the comparison between FEBID and FIBID, the significant difference in resistivity must be explained by all the features (atomic amount, size and distribution, and spacing between Pt particles), according to HRTEM and EDX analysis. At this point, it should be mentioned that the FEBID nanobeams also have an increased metallic content since gallium ions have been implanted by ion beam patterning. Therefore, in order to classify the results relative to other published values, this fabrication process must be considered.

### 3.3. Scanning Tunneling Microscopy

**Figure 9:**
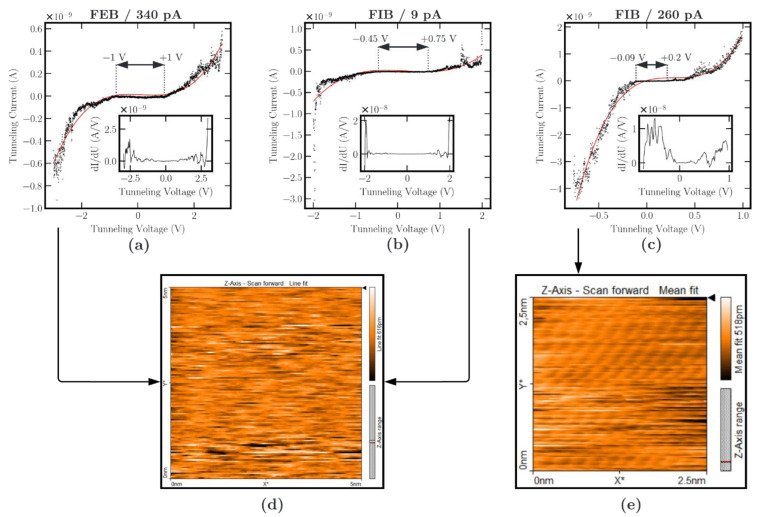
Results from scanning tunneling spectroscopy and microscopy measurements. Top: current-voltage curves from tunneling spectroscopy with fit function (red) and insets of the differential tunneling conductance dI/dU: (**a**) 340 pA FEB, (**b**) 9 pA FIB, (**c**) 260 pA FIB. Bottom: (**d**) imaging of the HOPG sample by scanning with the electrode tip from (**a**,**b**). (**e**) Image of a HOPG sample by scanning with the electrode tip from (**c**).

The application and investigation of the PtC tips with STM show significant differences in the required tunneling voltage per PtC electrode due to different band gaps of the PtC composite or edge layer thicknesses of the DLC. As expected from previous investigations in the present work, tips based on high ionic currents show significantly lower band gaps. Figure 9 shows the results in detail for three electrode tips for the different beam parameters. First, the band gap is visible from the tunneling spectroscopy, and second, the image of the HOPG sample shows the quality of the tunneling current based on the electrode composition. The characteristic of the I/U (current/voltage) measurement and its fit function by a third-order polynomial function shows the typical nonlinear tunneling behavior of a semiconductor. This is due to the conductance of zero in the region of the Fermi level. Doping of semiconductors can decrease the band gap and thus change the density of states at the Fermi level. Moreover, as shown by Pavlov et al. [52], decreasing the tunneling gap leads to a linearization of the I/U characteristic or shortening of the band gap. Both properties are achieved in this work by using the FIB and increasing the aperture current. The STS measurement from Figure 9a–c, therefore, shows that with an increasing aperture current, there is an increasing approach to the metallic behavior, since the band gap is shortened more and more due to:

- Increasing the metallic fraction according to the EDX (Figure 6) analysis;
- Decreasing the distance between the metallic particles in the microstructure, which is visible by lowering the resistivity, according to the RM (Figure 7) and the HRTEM analysis (Figure 8).

Therefore, the total distance of Pt particles, DLC layers, the vacuum gap, and the HOPG surface drops by increasing the beam current. Thus, a high beam current leads to an increase in tunneling suitability. This also becomes clear by comparing the differential tunneling conductance of the FEB and the FIB nanotips (insets in Figure 9a–c). The I/U measurements are very noisy due to the spatial expansion of the nanotip and the irregular arrangement of the Pt particles. Therefore, the derivatives of the I/U characteristic were smoothed by a Savitzky–Golay filter (filter window = 11, order of the polynomial = 3). According to Bardeen’s formula [53], the density of states correlates with the differential tunneling conductance dI/dU [51]. The differential tunneling conductance curves in Figure 9a–c confirm the characteristics of the semiconductor behavior and the shorting of the band gap by using the FIB and increasing the beam current. The band gap of the 340 pA FEB tip is marked by a voltage range of +/−1 V. Using the 9 pA electrode leads to a reduced band gap of −0.45 to +0.75 V. At a further increase of the used aperture current to 260 pA, there is a significant decrease in the band gap to the range of −0.09 to +0.2 V. In addition, the barrier height is unknown, since the tunneling spacing z was not determined, but can be identified as a function of the variation of the tunneling current, voltage, and spacing according to Olesen et al. [54].

Since this study aims to demonstrate tunneling using low tunneling voltages, the limit of the tunneling voltage can be determined by this measurement depending on the PtC tip used. Thus, the higher is the aperture current chosen, the lower is the possible tunneling voltage between the electrodes. Regarding the attractive forces, they are of utmost importance for use in a sensor application involving moving electrodes. The sensitivity can also be read directly based on the current amplitude during spectroscopy. The values of the electrode tips from Figure 9a,b are below 1 nA despite the high tunneling voltage, indicating a higher tunneling distance (total distance of Pt particles, DLC layers, vacuum gap, and HOPG surface). In contrast, the current curve in Figure 9c shows currents above 1 nA despite a significantly lower tunneling voltage. The sensitivity of the tunneling current is significantly higher here, indicating a lower tunneling distance. In contrast to low aperture currents or the use of the electron beam, this allows the sample’s atomic structure to be imaged with the tips of a 260 pA ion current using small tunneling voltages (500 mV). This is an elementary finding for the application of PtC electrodes in sensor structures, and it confirms the expectations from previous analyses (EDX and resistivity measurements).

## 4. Discussion

Examination of PtC by EDX analysis reveals significant differences in elemental content depending on the selected technology and aperture currents of FEB and FIB. Furthermore, the determination of resistivity and STM analysis allows for the comparison of electronic parameters. Thus, the results from the chosen investigation methods show which parameters of the dual beam are suitable for the implementation of the tunneling effect at low tunneling voltages. The main findings are discussed below.

The EDX analysis confirms the larger Pt content in the tunneling tips due to FIBID compared with FEBID. Moreover, the Pt amount increases further with a larger aperture current. However, the variation of the accelerating voltage and the dwell time shows no significant differences in the elemental amounts. Thus, as a result of the technology, FIBID PtC has a 14–20% higher metal content in the microstructure due to the ion implantation of Ga+, which further increases the tunneling suitability.

In addition, the resistivity of FIBID PtC is about two to three orders of magnitude higher, and that of FEBID PtC is about four orders of magnitude higher compared with the resistivity of pure Pt. The analysis shows that the resistivity increases with the reduction of the aperture current in FIBID. Looking at the EDX analysis, this is in line with the expectations. However, the higher material resistivity of FEBID PtC increases with an increasing aperture current. According to the HRTEM analysis (Figure 8), by increasing the aperture current, FEBID produces an opposing microstructure to FIBID. This leads to the hypothesis of low resistance with the most homogeneous possible distribution of small Pt particles with small spacing. This is due to the conduction mechanism of metal–organic materials corresponding to intrinsic tunneling.

The STM analysis also proves the tunneling effect with PtC tips. For this purpose, the imaging of a HOPG sample is investigated. The atomic structure of the graphene layer can be imaged at low tunneling voltages only with the 260 pA PtC tip. The spectroscopy measurements or the I/U characteristics of a 340 and 9 pA FEB tip and a 260 pA FIB tip demonstrate large differences in the band gap, in the sensitivity of the tunneling current, and in characteristics comparable to a semiconductor. At a larger aperture current (260 pA) using the FIB, the band gap decreases to a value between 90 and 200 mV. In contrast, the band gap for the FEB tip is about +/−1 V.

## 5. Summary

In summary, for a suitable configuration of the PtC tips to realize the tunneling effect at low tunneling voltages, the following points can be noted:

- The platinum content must be as high as possible (Figure 6).
- The platinum particles must have the smallest possible size and distance to each other. In the following, the distribution of the particles has a high density because large particles lead to larger spacings and thus higher material resistance. With a similar platinum content, according to EDX analysis (Figure 6), the PtC with smaller particle sizes, based on the HRTEM analysis (Figure 8), shows a lower resistivity.
- For the production of suitable PtC electrodes, the analyses show the following two essential requirements to decrease the electrode band gap (according to STS, Figure 9) and increase the tunneling suitability:
  ○ The use of FIBID instead of FEBID;
  ○ The use of the highest possible aperture current (260 pA) for FIBID.

The measuring range of the tunneling effect depends on the electrode material and the tunneling voltage. As shown in Section 2.1 and Section 3.2, the metal–organic material leads to high material and tunneling resistance, thus limiting the tunneling current or the measuring range. The dependence of the tunneling voltage results in different tunneling current amplitudes due to the linear relationship between tunneling current and voltage. Due to the high resistance corresponding to the application in STM, the tunneling current assumes a maximum value of a few nA depending on the tunneling voltage. Thus, the imaging of the HOPG sample provides evidence for the tunneling suitability of the PtC tips at low tunneling voltages as long as they are fabricated with FIBID using high aperture currents (260 pA).

## Figures and Tables

**Table 1:** Geometrical data and resistance measurements. From these measured values for the geometry of the nanowires, the total resistance, the resistance of the wiring, and the resistivity of the PtC can be calculated. Furthermore, the atomic amount of C, Ga, and Pt based on the EDX analysis (nanotips) is shown.

Current/Beam	R_w_ (Ω)	R_total_ (Ω)	Length (nm)	Width (nm)	Height (nm)	ρ_PtC_ (Ω·μm)	C (%)	Ga (%)	Pt (%)
9 pA IB	2547	2847	292	400	380	156.61	69.54	8.37	22.09
46 pA IB	937	1090	390	557	480	104.93	67.76	9.65	22.59
90 pA IB	667	785	440	570	516	78.86	66.91	9.23	23.86
260 pA IB	454	555	607	650	370	40.15	62.89	11.15	25.97
43 pA EB	1700	2962	428	330	525	510.69	-	-	-
340 pA EB	743	3273	543	370	607	1042.57	83.63	-	16.37
2.7 nA EB	1192	3433	393	400	680	1551.02	81.87	-	18.13

## Data Availability

All data used are shown in the text. Raw data are available on request.

## Abbreviations

The following abbreviations are used in this manuscript.

C	carbon
DLC	diamond-like carbon
EDX	energy dispersive X-ray spectroscopy
EELS	electron energy loss spectroscopy
FIB	focused ion beam
FEB	focused electron beam
FIBID	focused ion beam induced deposition
FEBID	focused electron beam induced deposition
GIS	gas injection system
Ga	gallium
HRTEM	high-resolution transmission electron microscopy
HOPG	highly oriented pyrolytic graphite
I/U	current/voltage
MEMS	microelectromechanical system
PCB	printed circuit board
Pt	platinum
PtIr	platinum iridium
PtC	platinum carbon
PolySi	polysilicon
RM	resistance measurement
SEM	scanning electron microscopy
SMU	source measuring unit
Si_3_N_4_	silicon nitride
STM	scanning tunneling microscope
STS	scanning tunneling spectroscopy
TEM	transmission electron microscopy
XPS	X-ray photoelectron spectroscopy
